# Sleep Dissolves Illusion: Sleep Withstands Learning of Visuo-Tactile-Proprioceptive Integration Induced by Repeated Days of Rubber Hand Illusion Training

**DOI:** 10.1371/journal.pone.0085734

**Published:** 2014-01-17

**Authors:** Motoyasu Honma, Takuya Yoshiike, Hiroki Ikeda, Yoshiharu Kim, Kenichi Kuriyama

**Affiliations:** Department of Adult Mental Health, National Institute of Mental Health, National Center of Neurology and Psychiatry, Tokyo, Japan; Centre de Neuroscience Cognitive, France

## Abstract

Multisensory integration is a key factor in establishing bodily self-consciousness and in adapting humans to novel environments. The rubber hand illusion paradigm, in which humans can immediately perceive illusory ownership to an artificial hand, is a traditional technique for investigating multisensory integration and the feeling of illusory ownership. However, the long-term learning properties of the rubber hand illusion have not been previously investigated. Moreover, although sleep contributes to various aspects of cognition, including learning and memory, its influence on illusory learning of the artificial hand has not yet been assessed. We determined the effects of daily repetitive training and sleep on learning visuo-tactile-proprioceptive sensory integration and illusory ownership in healthy adult participants by using the traditional rubber hand illusion paradigm. Subjective ownership of the rubber hand, proprioceptive drift, and galvanic skin response were measured to assess learning indexes. Subjective ownership was maintained and proprioceptive drift increased with daily training. Proprioceptive drift, but not subjective ownership, was significantly attenuated after sleep. A significantly greater reduction in galvanic skin response was observed after wakefulness compared to after sleep. Our results suggest that although repetitive rubber hand illusion training facilitates multisensory integration and physiological habituation of a multisensory incongruent environment, sleep corrects illusional integration and habituation based on experiences in a multisensory incongruent environment. These findings may increase our understanding of adaptive neural processes to novel environments, specifically, bodily self-consciousness and sleep-dependent neuroplasticity.

## Introduction

Mental representations of body orientation and configuration are a fundamental aspect of self-consciousness [1] and depend on afferent and efferent information about ongoing sensorimotor processes [2]. The integration of such multimodal sensorimotor information plays a crucial role in reciprocal reinforcement between body ownership and sense of belonging of afferent information [3], [4]. Recently, various aspects of multimodal sensorimotor integration that contribute to body ownership have been elucidated for vision, touch, pain, hearing, balance, gravity, and position sense (proprioception) [5]–[7], and integration is recognized as an important cognitive process for adapting to novel environments [8], [9].

The feeling of limb ownership is evoked by the multisensory integration of vision, touch, and proprioception, and has been postulated to be acquired a posteriori: young infants show consistent proprioceptive-visual invariants [1], [10], and adults can acquire the feeling of ownership of an artificial limb by repetitive multisensory learning [11]. Under this integration process, vision dominates over touch and proprioception [12], [13]; therefore, artificially displaced images of a limb simultaneously provided with illusory sensations of touch and proprioception elicit the feeling of limb ownership [11] via adaptively configured vision-touch and vision-proprioception integrations. Such visually mismatched multimodal integration has been investigated in humans since the 19th century with prismatic glasses [14]. At the beginning of prism exposure, subjects produce endpoint errors in optical shift direction when pointing to a visual target. However, subjects gradually adapt their motor commands until they achieve accurate movements within a few days, suggesting that configuration of sensory integration between vision and other modalities, including vision-proprioception integration, takes place over days. However, the long-term regulation of illusory limb ownership consolidation has not been elucidated.

Such adaptive skill training universally elicits both immediate (online) skill improvement and delayed (offline) skill consolidation processes. It has been established that sleep substantially contributes to offline skill consolidation, probably by enhancing neural plasticity contributing to various skill consolidation processes [15]–[17]. The contribution of sleep to skill consolidation is demonstrated by skill enhancements after post-learning sleep, but not after a similar period of wakefulness [18]–[20].

Here, we examined the effect of daily repetitive training (in Experiment 1) and post-training sleep (in Experiment 2) on artificial limb ownership learning by means of the rubber hand illusion (RHI) paradigm [11], [21], [22] on the basis of proprioceptive drift (PD) [11] and changes in physiological arousal [23] that contribute to the development of the feeling of artificial limb ownership [24], [25]. We hypothesized that sleep may boost PD and decrease physiological arousal for long-term development of illusory limb ownership.

## Materials and Methods

### Participants

Procedures were performed according to the ethical guidelines in the Declaration of Helsinki. The Intramural Research Board of the National Center of Neurology and Psychiatry approved the study protocol, and all participants provided written informed consent. Fourteen healthy, right-handed young adults (mean ± standard error of the mean [SEM], 21.1±0.25 years; range, 20–23 years; 5 female) and 38 healthy, right-handed young adults (21.4±0.29 years; range, 20–24 years; 15 female) participated in experiments 1 and 2, respectively. They reported no previous history of drug or alcohol abuse or neurological, psychiatric, or sleep disorders and maintained a constant sleep schedule. They were instructed to remain drug-, alcohol-, and caffeine-free for 24 h before and during the study period.

### Experimental settings

**Figure 1:**
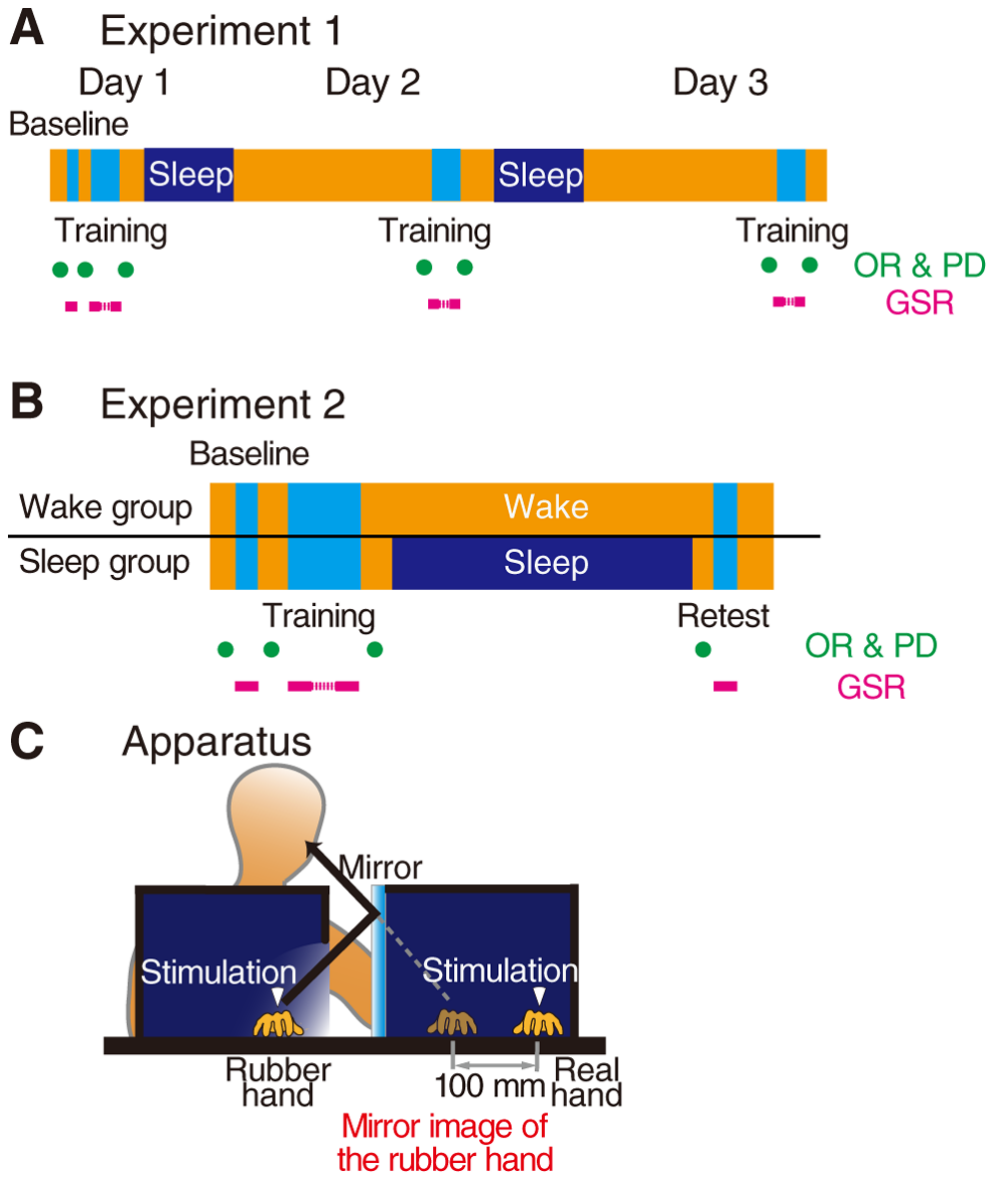
Experimental paradigm: Schematic representation of consecutive baseline and training sessions and the delayed retest session. (A) Experiment 1 consisted of a baseline session and 3 daily repetitive training sessions at 24-h intervals for 14 healthy participants. (B) Experiment 2 consisted of baseline and training sessions, and a retest session 12 h after the training session for 38 healthy participants randomly allocated to the wake and sleep groups. (C) During training and retest, the participant's real hand was draped, and the mirror image of a realistic life-sized rubber hand (RH) was reflected 100 mm to the right of their real hand. Both the rubber and real hands were gently touched with synchronized 0.5-Hz sticks. The training and retest sessions consisted of 150 and 15 stimulation repetitions, respectively. During the baseline session, only the participant's real hand was touched 15 times. The subjective ownership rating of the rubber hand (OR) and proprioceptive drift (PD) were repeatedly measured immediately before and after each baseline, training, or retest session. Physiological/autonomic responses were simultaneously measured by the galvanic skin response (GSR) during these test sessions.

To investigate the effect of daily repetitive training on acquiring the feeling of limb ownership of an artificial hand, 14 participants were repetitively trained with the RHI paradigm for 3 consecutive days after a baseline assessment session (Experiment 1). After performing their normal daily activities at home, they came to our laboratory and were trained at 8:00 PM and then re-trained twice after 24 and 48 h on consecutive days (Fig. 1A).

To investigate the effect of post-training sleep on consolidating the feeling of limb ownership, we randomly formed two equal-sized groups (Experiment 2), thus eliminating biases for age (*p*>.1) and sex (*χ^2^* = 0.11, *p* = .74). Participants spent the entire semidiurnal experimental period in the laboratory regardless of their sleep and wake period assignments (Fig. 1B). Those in the sleep group were trained at 8:00 PM and retested at 8:00 AM the next morning after a night of sleep (7.15±0.16 h, confirmed by an ambulatory wrist activity recorder), and those in the wake group were trained at 8:00 AM and retested at 8:00 PM after a 12-h wake interval. Baseline performance was assessed before RHI task training.

### RHI task

During training and retesting, participants received tactile stimulation on their left hand with a wooden stick (150 and 15 repetitions, respectively) while viewing synchronized stimulations of a realistic life-sized rubber hand (RH) via a mirror attached to a black box (see also, [22], [26]). The participant's real hand on the table was covered with a black box, and the RH was reflected in a mirror 100 mm to the right of the real hand (Fig. 1C). Both the RH and the participant's hidden real hand were gently pricked with sticks (frequency, 0.5 Hz), the timing of which was synchronized by computer control. Just before training, baseline assessment was performed by stimulating the participant's real hand (covered with a black box) 15 times with a stick, without any stimulation performed on the RH.

We assessed the ownership rating (OR) of a RH with a 100-mm horizontal visual analog scale (VAS) labeled “I did not feel ownership of the RH” on the left extremity and “I felt as if the RH were my hand” on the right extremity. Participants were asked to draw a vertical mark on the line at the point corresponding to their present status 1 min before and after baseline and training and before retesting as a subjective measure of illusory ownership. A higher OR score is associated with a greater sense of RH ownership [11]. We simultaneously measured the felt position of the hidden real hand, which is an objective measure of PD reflecting the visuo-tactile-proprioceptive integration level, and greater PD is associated with greater multisensory integration [21]. With their eyes closed, the participants were asked to draw, with their right index finger, a straight line on the opposite side of the table until it was judged to be in alignment with the middle finger of the left hand, which rested on the table in the same position as during training. During the first and last 30 s of training and throughout the baseline and retest periods, we measured galvanic skin response (GSR) in microsiemens (µS), a unit of electrical conductance, to determine the impact of newly acquired multimodal integrative learning on physiological/autonomic regulation [11], [23]. This is an important biomarker that corresponds to adaptive acquisition to novel environments [27], [28] and has also been shown to be a reliable objective index of illusory limb ownership [24], [25]. Previous RHI studies utilized GSR as a measure of the strength of visuo-tactile-proprioceptive integration at a perceptual level similar to PD [24], [25], [29]. However, we regarded it as measure of incongruence between higher cognitive and perceptual hand ownership in this study, because most previous studies measured event-related GSR when an aversive stimulus was applied to the RH [24], [25], [29], and greater visuo-tactile-proprioceptive integration of RHI at a perceptual level enhanced physiological fear in response to the threat stimuli to the RH. In contrast, the current study measured it during the simple RHI learning phase without any additional threat stimuli to the RH. Thus, the current GSR may reflect physiological arousal levels activated by the robust incongruence between cognitive and perceptive levels of RHI, similar to the oddball GSR [30]. According to our hypothesis, physiological arousal may show habituation with the consolidation of visuo-tactile-proprioceptive integration. Two electrodes filled with an isotonic electrolyte (0.05 M NaCl) were attached to the palmar surface of the annular and little fingers of the right hand. GSR was sampled at 250 Hz, and the event-specific GSR amplitude was calculated by subtracting the minimal value from the peak value in each trial [23]. Thereafter, the mean GSR value was computed for each baseline, pre-training, post-training, and retest periods.

### Statistics

Two-way analyses of variance (ANOVA) were applied to repeated measures of behavioral indexes (OR, PD, and GSR) relative to baseline values for detecting within (pre-training–post-training) and across (Days 1–3) training effects, as well as the effect of their interaction on RHI development (Experiment 1). To test the possible interval effect in RHI development, paired *t*-tests were used to compare changes in behavioral indexes (ΔOR, ΔPD, and ΔGSR) calculated by subtracting the value at the post-training point from that at the pre-training point of the previous day (e.g., ΔOR_1st ITI_  =  OR_(pre-training on day 2)_ − OR_(post-training on day 1)_) during the first and second 24-h inter-training intervals (ITIs).

Two-way ANOVAs were applied to repeated measures of behavioral indexes (OR, PD, and GSR) compared to baseline values to detect training – retest (pre- and post-training – retest) and group (sleep vs. wake) effects, as well as the interaction between them (Experiment 2). Independent samples *t*-tests were used to compare group differences of change in behavioral indexes (ΔOR, ΔPD, and ΔGSR) during 12-h training – retest intervals. To examine a possible reciprocality among PD, physiological arousal, and the feeling of RH ownership, Pearson's correlation coefficients were calculated for both immediate training (e.g., ΔOR_immediate_  =  OR_(post-training)_ − OR_(baseline)_) and delayed learning (e.g., ΔOR_delayed_  =  OR_(pre-retest)_ − OR_(post-training)_) effects in each group. ANOVAs were followed by post-hoc tests. Results are shown as the mean ± SEM. The level of statistical significance was defined as *p*<0.05 after Bonferroni correction for multiple comparisons. When *p* values between .05 and .10 were obtained, although nonsignificant, we considered that this suggested a likelihood or trend toward significance.

## Results

### Experiment 1: Daily repetitive training effects on RHI development

**Figure 2:**
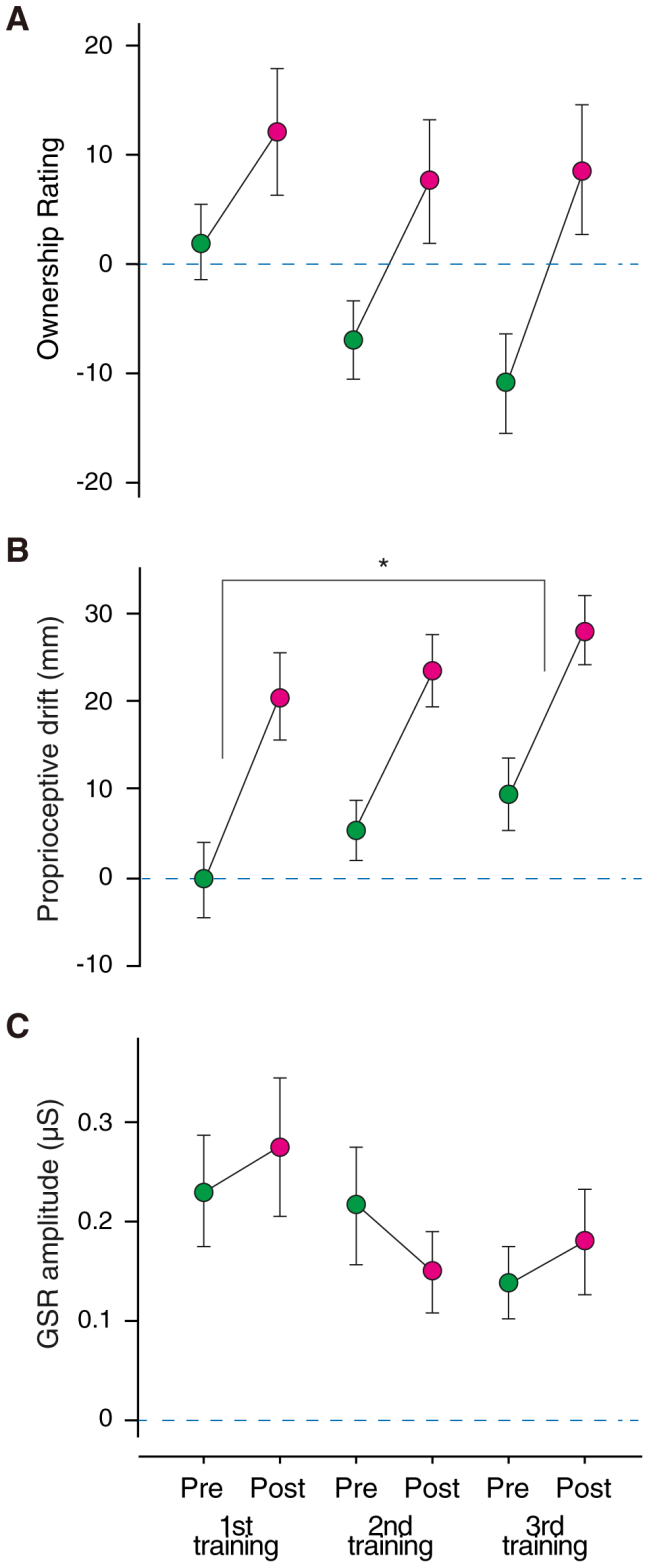
Results of Experiment 1: Time course of the developments (A–C) of the subjective ownership rating of rubber hand (OR), proprioceptive drift (PD), and galvanic skin response (GSR) during RHI training session on all 3 experimental days. The dashed lines indicate the mean baseline levels of OR, PD, and GSR. The green and pink circles indicate the mean changes in OR, PD, and GSR from baseline to the pre- and post-training periods, respectively. Error bars indicate the standard errors of the mean (SEM). *trends by post-hoc test against the pre-training point.

At the beginning of the baseline session, participants immediately acquired the feeling of illusory ownership for the RH (OR = 47.0±2.60) upon first viewing the RH set on the virtually matched point (although an individual training seemed to facilitate OR, repetitive RHI training did not). A two-way ANOVA showed a significant within-training effect (*F*(1,78) = 13.3, *p* = .0005) but nonsignificant across-training effect (*p* = .220) and interaction (*p* = .639) on OR. Post-hoc tests revealed a significantly higher OR at post-training than at pre-training (*p* = .0005; mean ΔOR = 14.1; Fig. 2A). OR decreased equally during the first and second ITIs. Paired *t*-tests revealed no significant differences between ΔOR (−18.2±6.09) during the first ITI vs. ΔOR (−17.7±5.27) during the second ITI (*t* = −0.102, *p* = .921).

In contrast, although participants' reaches were already displaced (12.0±2.98 mm) rightward toward the RH as a result of methodological artifacts even before the baseline session, the within-training effect was observed in PD. A two-way ANOVA showed a significant within-training effect (*F*(1,78) = 31.9, *p*<.0001) and a trend toward an across-training effect (*F*(2,78) = 3.24, *p* = .075), but a nonsignificant interaction (*p* = 0.946) on PD. Post-hoc tests revealed significantly greater PD at post-training compared to pre-training (mean ΔPD = 19.1 mm; Fig. 2B), and suggested greater PD during the third training session compared to the first (mean ΔPD = 8.57 mm; *p* = .042). PD also decreased equally during the first and second ITIs. Paired *t*-tests revealed no significant differences between ΔPD (−1.52±0.423) during the first ITI vs. ΔPD (−1.40±0.283) during the second ITI (*t* = −0.296, *p* = .772).

The mean baseline GSR value was 0.11±0.025 µS. GSR increased immediately at the beginning of RHI training on the first day and remained high throughout the experiment. Two-way ANOVA did not show any significant training effects or interaction for GSR (all *p*>.10, Fig. 2C). GSR did not seem to show any changes during the ITIs. Paired *t*-tests showed no significant differences between ΔGSR (−0.0576±0.0974) during the first ITI vs. ΔGSR (−0.0111±0.0463) during the second ITI (*t* = −0.470, *p* = .647).

### Experiment 2: Effects of post-training sleep on RHI development

**Figure 3:**
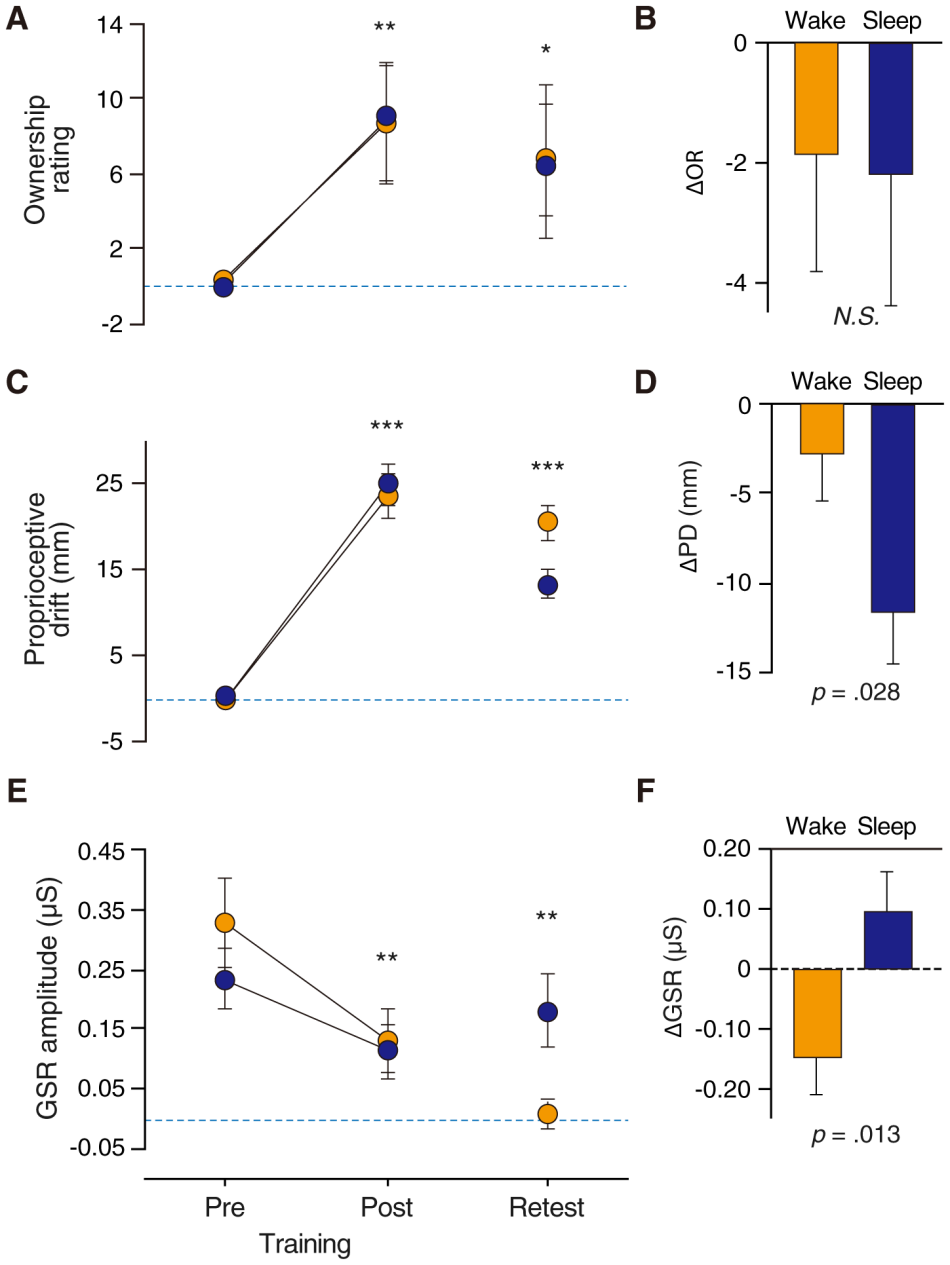
Results of Experiment 2: Immediate changes during daytime training sessions (A, C, and E in the left column) and delayed changes during the 12-h training-retest interval (B, D, and F in the right column) of the subjective ownership rating of rubber hand (OR), proprioceptive drift (PD), and galvanic skin response (GSR) during RHI training and retest. The dashed lines indicate the mean baseline levels of OR, PD, and GSR. The points (yellow circle, wake group; blue circle, sleep group) and error bars indicate the mean changes in OR, PD, and GSR and the standard errors of the mean (SEM) from baseline to the pre-training, post-training, and retest points. ***p<.0001, **p<.005 and *trends by post-hoc test against the pre-training point. The yellow and blue bars with error bars indicate the mean values (and SEM) of OR, PD, and GSR changes (retest – post-training) in the wake and sleep groups, respectively, during the 12-h training-retest interval.

At the beginning of the baseline session, participants immediately acquired the feeling of illusory ownership for the RH (OR = 8.92±1.43), but OR showed minimal changes after the baseline trials (0.29±0.21). After training, participants reported greater RH ownership (8.78±3.17; Fig. 3A). A two-way ANOVA revealed a significant training – retest effect (*F*(2,108) = 5.15, *p* = .007), but did not show any group effects (*p* = .994) or training – retest × group interaction (*p* = .998) on OR. Post-hoc tests revealed significantly greater OR at post-training compared to pre-training (*p* = .003), and marginally greater OR at retest than at pre-training (*p* = .063; Fig. 3A). A two-sample *t*-test for ΔOR showed a nonsignificant difference between the sleep and wake groups (*t*(36) = −0.11, *p* = .916; Fig. 3B), suggesting that OR decreased slightly in both groups during the training – retest interval (wake group: −1.87±1.98, group: −2.18±2.22).

At the beginning of the baseline session, participants' reaches were also displaced (PD = 36.2±2.08 mm) rightward toward the RH, and maintained that level after the baseline trials (0.08±0.45 mm). After training, participants' reaches were displaced rightward (24.4±1.71 mm) toward the RH (Fig. 3C). A two-way ANOVA revealed a significant training-retest effect (*F*(2,108) = 92.1, *p*<.0001) and a significant training – retest × group interaction (*F*(2,108) = 3.21, *p* = .044), but did not show any group effects (*p* = .216) on PD. Post-hoc tests showed significantly greater PD at post-training than at pre-training (*p*<.0001) or retest (*p* = .0001), and significantly greater PD at retest than at pre-training (*p*<.0001; Fig. 3C). A two-sample *t*-test for ΔPD showed a significant group difference between the sleep and wake groups (*t*(36) = 2.30, *p* = .028; Fig. 3D), suggesting that PD in the sleep group was significantly displaced leftward toward the real hand, nearing the initial position (−11.7±2.89 mm), whereas PD in the wake group remained at almost the same position (−2.95±2.48 mm) as during the training – retest interval.

GSR showed small changes during the baseline session (0.098±0.010). GSR quickly increased (0.28±0.062 µS) at the initial stage of training but decreased at a later stage (−0.16±0.012 µS), suggesting that physiological arousal decreases as artificial limb ownership sensation matures. A two-way ANOVA revealed a significant training-retest effect (*F*(2,108) = 7.19, *p* = .001) and a significant training − retest × group interaction (*F*(2,108) = 3.35, *p* = .039), but no group effects (*p* = .645) on GSR. Post-hoc tests showed significantly higher GSR at pre-training than at post-training (*p* = .003) or retest (*p* = .0006; Fig. 3E). A two-sample *t*-test for ΔGSR showed a significant group difference between the sleep and wake groups (*t*(36) = 6.89, *p* = .013; Fig. 3F), suggesting that GSR was drastically reduced in the wake group (−0.15±0.070 µS), whereas it was increased in the sleep group (0.10±0.070 µS) during the training – retest interval.

**Figure 4:**
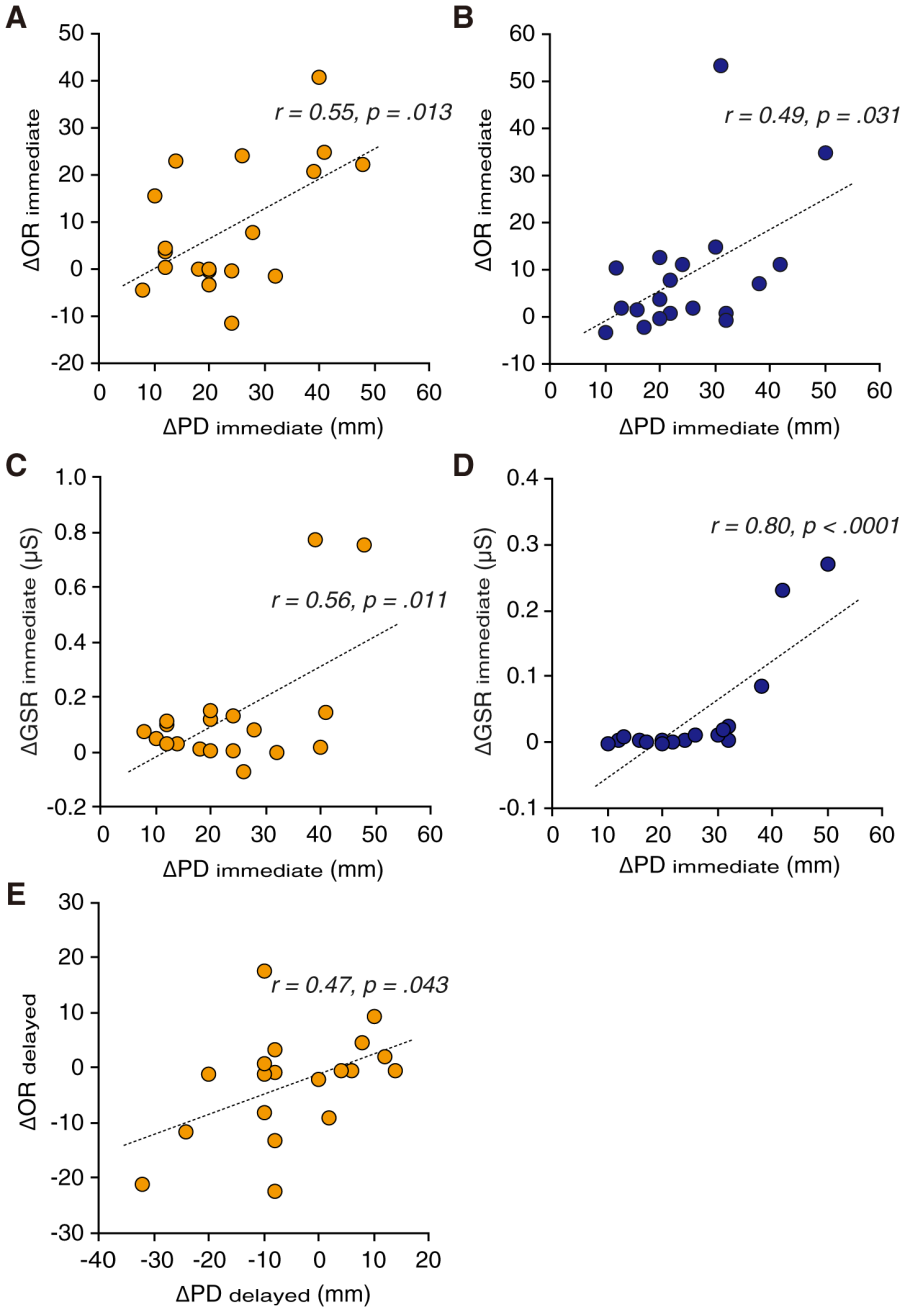
Relationship between changes in proprioceptive drift (ΔPD), changes in the subjective ownership rating of rubber hand (ΔOR), and changes in galvanic skin response (ΔGSR) with regression lines. The yellow and blue circles indicate values in the wake and sleep groups, respectively. ΔOR during immediate training was significantly correlated with ΔPD during immediate training in both the wake (A) and sleep (B) groups. ΔGSR during immediate training was significantly correlated with ΔPD during immediate training in both the wake (C) and sleep (D) groups. However, ΔOR during delayed learning was only significantly correlated with ΔPD during delayed learning in the wake group (E).

Although PD typically contributed to the feeling of artificial limb ownership, particularly during the immediate training process, physiological arousal (GSR) did not directly contribute to artificial limb ownership during immediate training or delayed learning processes. Pearson's correlation analyses showed significant correlations between ΔPD_immediate_ and ΔOR_immediate_ in the wake (*r* = 0.55, *p* = .013; Fig. 4A) and sleep (*r* = 0.49, *p* = .031; Fig. 4B) groups. However, although PD contributed to ownership of the artificial limb after the 12-h interval of wakefulness, it no longer contributed to ownership after the 12-h interval of sleep; no significant correlation between ΔPD_delayed_ and ΔOR_delayed_ was found for the sleep group (*p*>.05), but it was evident in the wake group (*r* = 0.47, *p* = .043; Fig. 4E). These findings suggest that sleep weakens relationships between OR and PD by enhancing exclusively to PD. Physiological arousal seemed to associate with PD during the immediate training period regardless of group. Significant correlations were observed between ΔPD_immediate_ and ΔGSR_immediate_ in the wake (*r* = 0.56, *p* = .011; Fig. 4C) and sleep (*r* = 0.80, *p*<.0001; Fig. 4D) groups. However, no significant correlations were observed between ΔPD_delayed_ and ΔGSR_delayed_ in either group (all *p*>.05). Thus, physiological arousal may just be a minor reaction contingent to PD.

## Discussion

### Accumulated training effects on artificial limb ownership

Although individual RHI training sessions immediately resulted in greater illusory ownership and greater visuo-tactile-proprioceptive integration in line with previous reports [11], [21], [31], daily repetitive RHI training simply maintained illusory ownership, but potentially guided toward continuous improvement in visuo-tactile-proprioceptive integration. OR and PD were consolidated during the first training period and were strongly diminished during the ITIs, but they were reestablished by the post-ITI trainings. It seems possible that longer or more frequent training can elicit a significant across-training effect in PD. This is the first report showing an accumulated training effect of RHI and a discrepancy between illusory ownership and visuo-tactile-proprioceptive integration.

However, considering the fact that participants with illusory limb ownership still experience the original location of their own hand via afferent proprioceptive signaling, participants may have to discriminate between their own hands and the RH while continuing to develop multisensory (visuo-tactile-proprioceptive) integration. In fact, humans subjected to a similar visually mismatched multisensory integrative learning paradigm (prism exposure) continue to adapt to the real visually matched environment without prism glasses after they adapt to the novel mismatched environment [13], [14]. The feeling of artificial limb ownership is associated with activity in the ventral premotor cortex (VPMC), inter parietal sulcus (IPS), insula, and sensorimotor cortex [31]–[33]. As with tool use [34], [35], visuo-spatial receptive fields in the IPS and/or VPMC may be remapped (shifted or enlarged) to simultaneously accept artificial hand position along with a rake-like extension of the hand [36]. Makin, Holmes, & Ehrsson [37] proposed that neural reorganization might also occur with RHI. The behavioral alterations in OR and PD may reflect the consolidation of such neuroplastic changes in the IPS and/or VPMC. A recent study [38] suggested that different cortical areas were linked to OR and PD. Remapping in the VPMC was associated with the change in OR, and that in the posterior parietal cortex (including the IPS) was associated with the change in PD [38]. These independent functional associations between regional activation and behavior possibly enable discrete changes in OR and PD. In addition, the premotor cortex and the lateral occipital cortex were proposed as the key regions for consolidating subjective hand ownership and physiological responses to the involvement of an RH [29].

Contrary to our hypothesis, GSR seemed to peak during the initial training period and was then maintained during subsequent training and ITIs. GSR during the RHI task may reflect a threat or strangeness directed toward the RH caused by a mismatch between higher cognition (orientation) and perceptual (visuo-tactile-proprioceptive) integration; thus, GSR changes correlated well with changes in PD during the immediate training phase. Such threat or strangeness may be generated from conflicts between the higher cognitive and perceptual levels of RHI, thus the greater increase in PD is accompanied by the greater increase in GSR. Previous reports suggested that GSR to the invasive stimulation of the RH reflects illusory hand ownership [24], [25]. Clinical studies also paradoxically support the hypothesis that amputated patients with phantom pain exhibit greater decrement in physiological response to aversive stimuli associated with pain perception when they adjudicated a visuo-tactile conflict and established virtual body ownership of the stimulated hand [23], [39]. Although cumulative training seemed to shift PD toward the RH, GSR did not show any significant immediate or accumulating training effects in the current study. Combined with our results, GSR represents an aspect of a long-term goal of illusory learning; GSR may correspond to illusory learning immaturity, and if the incongruence between the cognitive and perceptual levels of RHI could resolved (one has perceived the RH to be absolutely one's own hand), GSR would decrease toward the baseline level.

### Effects of post-training sleep on artificial limb ownership

Individual RHI training temporarily enhanced OR and PD, but OR and PD were almost diminished to baseline levels during ITIs. GSR was enhanced at the beginning of the first RHI training and remained elevated during the entire experimental period, regardless of the course of RHI training or ITI. Although sleep does not seem to affect artificial limb ownership per se, it may modulate the delayed consolidation of artificial limb ownership by diminishing visuo-tactile-proprioceptive integration and preventing physiological arousal inhibition. In other words, sleep resets the proprioceptive sensation closer to reality and maintains awareness that the RH is an alien limb, which may reflect a vital role of sleep in the selective modulation of skill learning induced by repetitive RHI training. There are greater opportunities to correct PD by the actual visuo-tactile-proprioceptive congruence of one's own limb during wakefulness compared to sleep; however, greater correction in PD occurred during sleep rather than wakefulness. Besides, our results suggest that sleep preserved the physiological arousal associated with visuo-tactile-proprioceptive incongruence. Hence, sleep does not seem to haphazardly enhance memories [15]; rather, it seems to resist incongruent multisensory integration in an adaptive concept.

Sleep-dependent memory processing guides memory enhancements by selecting the most relevant information from its own autobiographical history and optimally integrates it into memory networks [40]. Two selection biases in sleep-dependent memory enhancement have been assumed. One is emotionality of the remembered experiences: more emotional experiences are consolidated by sleep-dependent memory processing, especially during rapid eye movement sleep [41]–[43]. The other bias is profitability of the remembered experiences: when greater recall performances provide greater rewards, memory is consolidated more by sleep-dependent memory processing [44], [45]. Because these two biases have not always occurred in unison with the current situation in which greater multimodal integration elicits greater emotionality and greatly eliminates profit in a short-term perspective, sleep-dependent memory processing may make a choice in accordance with a novel comprehensive adaptive nature, regardless of emotionality or profitability.

### Sleep leads to long-term change in RHI training-to-reality adaptation

It remains unknown why artificial limb ownership after sleep is felt to the same extent as after wakefulness, even though greater correction of PD occurred after sleep compared to a similar period of wakefulness. It is likely that there are at least two processes involved in RHI: acquiring limb ownership does not always require proprioceptive accordance with the real hand. It has been suggested that limb ownership is established by both a “bottom-up” process of visuo-tactile matching via afferent neural input integration [23], [46] as well as a “top-down” process of realistic acceptance at a higher cognitive level [47]. Greater bias in RH rotational position or shape considerably diminished PD [48], [49], suggesting that the “top-down” process of realistic acceptance contributes to the optimization of illusory ownership for successful adaptation. A long-term goal of illusory learning, including RHI, may optimize “dual-orientation” of ownership for the real and artificial limb positions: the artificial hand was perceived as an own limb without inducing significant disownership of the real hand. Similar cognitive architectures are often observed in schizophrenic delusions in which a thought or body is simultaneously attributed to both oneself and others. Indeed, patients with schizophrenia exhibited greater illusory ownership to the RH and greater PD [50], [51] due to greater “top-down” modulation for establishing “dual-orientation” of ownership for both the real and artificial hand, enough to overwhelm conflicting “bottom-up” sensation. Mirror visual feedback (MVF) therapy, which utilizes a similar method to that of the RHI technique, is one of the most effective treatments for phantom pain, i.e., chronic pain of central origin after limb loss [52]–[54]. Although its etiology is poorly understood, phantom pain is a response to discrepancies between different senses [55]. Repetitive MVF training works by restoring visual-proprioceptive congruence between the mirror-imaged contralateral limb and the phantom limb [56], [57]. The treatment effectively reduces pain, even though patients recognize, at a higher intellectual level, that the real limb has been lost and that the mirrored limb is an illusion. It also suggests that MVF training optimizes the “top-down” process distorted by the unacceptable event of limb loss. Given that repetitive administrations of the RHI and MVF result in a common learning goal, repetitive exposure to the RHI may simply facilitate “bottom-up” processing of visuo-tactile-proprioceptive integration, whereas sleep independently enhances “top-down” processing of cognitive optimization toward reality adaptation.

The synaptic homeostasis hypothesis assumes that memory consolidation is a by-product of global synaptic downscaling that occurs during sleep [58]–[60]. This hypothesis suggests that active synaptic downscaling occurring during sleep is beneficial for cellular efficiency and facilitates overnight delayed improvement of skill performances, and it implies that active synaptic downscaling occurring during sleep performs triage and directs a limited cellular margin to a high-priority skill and abandons low-priority skills based on the principle of adaptation. The current results provide support for this hypothesis in that sleep-dependent learning might enhance an element of skill while simultaneously attenuating another element. In this study, physiological arousal was robustly reflected in adaptive aspects of behavior to reality; thus, GSR appeared to be enhanced by the dynamism, adapting to real-world behavior while attenuating illusory proprioception during sleep [57]. The multisensory integration was maintained during wakefulness, while physiological habituation to the illusory hand phenomenon was inhibited during sleep. Thus, although the current results could represent yet another aspect of sleep-regulated memory modulation, it may actually reflect the nature of sleep.

### Limitations

Our findings are subject to a few potential limitations. First, we identified no concrete across-training effects during the three days of the experimental period. Previous reports suggest that long-term repetitive training of similar multisensory integration in a novel environment over the course of a month elicited greater adaptation to the environment [14]; thus, repetitive RHI training should also elicit greater multisensory integration. The current study induced the synchronized stimulation 150 times per day for 3 days. To confirm the value of the current results, future research should examine whether longer or more frequent RHI training could elicit a significant across-training effect. Second, our results could be confounded by a possible circadian effect. Although previous studies on sleep-dependent learning showed scant circadian influence on long-term development of various learning domains in humans [61], [62], it is unclear whether multimodal integration learning (including RHI training) would be affected by circadian regulation. Future studies should focus on the contributions of proprioception and physiological arousal to the feeling of artificial limb ownership, and determine which neural network(s) contribute to this type of learning during sleep. These goals could be achieved with a longitudinal neurophysiological study that simultaneously assesses potential circadian effects.
